# Evaluation of the effect of insulin sensitivity-enhancing lifestyle- and dietary-related adjuncts on antidepressant treatment response: protocol for a systematic review and meta-analysis

**DOI:** 10.1186/s13643-019-0978-8

**Published:** 2019-02-25

**Authors:** Olaitan J. Jeremiah, Gráinne Cousins, Finbarr P. Leacy, Brian P. Kirby, Benedict K. Ryan

**Affiliations:** 1 School of Pharmacy, Royal College of Surgeons in Ireland (RCSI), 123, St Stephen’s Green, Dublin 2, Ireland; 2 Data Science Centre, Royal College of Surgeons in Ireland (RCSI), 123, St Stephen’s Green, Dublin 2, Ireland

**Keywords:** Depression, Adjuncts, Insulin sensitivity, Lifestyle, Vitamin D, Zinc, Magnesium, Unsaturated fatty acids, Probiotics, Systematic review

## Abstract

**Background:**

Depression is the leading cause of disability worldwide and is known to be associated with insulin resistance (IR). Insulin resistance worsens the symptoms of depression and reduces the effectiveness of antidepressant medications in some depressed patients. Many studies have assessed the effect of adjunctive exercise, vitamin D supplementation, zinc supplementation, magnesium, probiotics, unsaturated fatty acids, and hygienic-dietary recommendations (sleep hygiene, healthy diet, physical activity, and sunlight exposure, combined or singly used), individually, on antidepressant treatment response. However, despite the reported insulin sensitivity-enhancing potential of these adjuncts, no systematic review has collectively analysed their antidepressant effect with regards to insulin sensitivity.

**Methods/design:**

In this systematic review, we will analyse the effect of the above-stated adjuncts on antidepressant treatment response (primary outcome) in comparison with treatment as usual with or without adjunctive placebo after identifying the relevant trials from a systematic literature search. Randomised controlled trials involving clinically depressed patients with diagnosis of major depressive, dysthymic or bipolar disorder will be considered. Changes in insulin sensitivity parameters, following treatment, will also be analysed as the secondary outcome. Effect estimates of the included trials will be combined using random-effects meta-analysis, while addressing risk of bias issues. Any significant heterogeneity between studies will be explored using sensitivity and subgroup analyses.

**Discussion:**

The findings of this review will contribute to the evidence base regarding the utility of non-pharmacological insulin-sensitising treatments in enhancing conventional antidepressant treatment response.

**Electronic supplementary material:**

The online version of this article (10.1186/s13643-019-0978-8) contains supplementary material, which is available to authorized users.

## Background

Depression affects more than 300 million people of all ages globally and has been identified as the leading cause of disability worldwide [1]. The characteristic depressed state, which occurs during major depressive episodes (MDE) in major depressive disorder (MDD) and bipolar disorder (BD), is a global health concern which poses a continuously rising socio-economic burden due to its association with disability, morbidity and mortality [2, 3]. Moreover, it has been noted that patients with PDD/dysthymia often present with MDE, and their response to antidepressant treatment is the same as in MDD patients [4]. Dysthymic disorder and chronic major depressive disorder, as defined in DSM-IV, have now been consolidated in DSM-5 as persistent depressive disorder (PDD)/dysthymia, hereafter referred to as dysthymia.

The main therapeutic goal in the treatment of depression is symptomatic remission. This is believed to increase the probability of complete patients’ recovery and restoration to their pre-disease state of mental health and psychosocial functioning [5]. Antidepressant treatment outcome measured in terms of percentage reduction in depression scores or absolute scores using standard depression rating scales is also usually considered in clinical trials. This is because it is a predictor of final response and remission [6]. Despite the recent progress in the prevention and detection of depression as well as treatment with currently available standard/conventional antidepressants (e.g. monoamine-based: fluoxetine, sertraline, citalopram, escitalopram, venlafaxine, reboxetine and mirtazapine) and mood stabilisers (lithium salts—commonly used in BD, atypical antipsychotics), only 60–70% of depressed patients respond to standard antidepressant treatment (treatment as usual (TAU)), leaving some depressed patients (at least 30%) with unsatisfactory or no response to therapy [5, 7]. The predominant depression pole of illness in BD also sometimes proves resistant to standard mood-stabilising therapy; hence, there remains an unmet need in the treatment of this condition [8]. In an attempt to overcome the problem of treatment resistance (failure to attain remission with at least two trials of adequate doses and duration of standard antidepressant treatment) in depression, the standard antidepressants are often augmented with other psychotropic medications such as atypical antipsychotics (e.g. asenapine, aripiprazole, olanzapine, quetiapine), anticonvulsants (e.g. carbamazepine, lamotrigine) and psychostimulant (lisdexamfetamine), with the use of atypical antipsychotics being the most extensively and rigorously used/studied pharmacological approach [5]. There is also evidence to support the use of metabolic interventions and immune-based treatments for depression, especially treatment-resistant depression (TRD). Non-pharmacological approaches in TRD treatment include somatic/neuromodulation therapies (e.g. electroconvulsive therapy) and behavioural therapy including aerobic exercise and cognitive behavioural therapy [5].

The complexity and heterogeneity of depression in terms of its etiopathogenesis suggest a need for mechanistically different but validated treatment interventions.

Growing evidence suggests that antidepressant treatment effectiveness may be influenced by insulin resistance. Recent population-based studies [9, 10] and systematic review [11] have identified an association between depression and insulin resistance as well as metabolic syndrome [12]. Evidence has also shown a bidirectional relationship between depression and type 2 diabetes mellitus [13]. Moreover, a study has recently shown that impaired insulin sensitivity frequently occurs in MDD patients [14], and there is evidence of improved insulin sensitivity in depressed (non-diabetic) patients following successful treatment and clinical remission [15]. Also, a recent review and a meta-analysis have shown the potential antidepressant effect of insulin sensitizers [16, 17]. Importantly, correction of insulin resistance, along with other related mechanisms, through the use of add-on insulin sensitizer therapy, has been demonstrated in clinical trials to improve antidepressant treatment response in patients with treatment-resistant depression [18, 19]. Lin et al. [18] found that pioglitazone (an insulin sensitizer) significantly reduced IR-associated antidepressant treatment resistance and also reported a positive correlation between improvement in glucose metabolism and improvement in depression scores (*r* = 0.51, *p* = 0.03; oral glucose tolerance test (OGTT) and HAM-D scores). A number of mediators have been suggested to be involved in the pathophysiological pathways linking insulin resistance with depression/depression treatment response. Most of these mediators have also been identified as key players in a multiple adaptive and survival-promoting processes called allostasis. These include glutamate and acetyl-L-carnitine (LAC), brain-derived neurotrophic factor (BDNF) and peroxisome proliferator-activated receptor gamma (PPAR-γ) [20]. For instance, glutamate-induced neurotoxicity contributes to dysfunctional brain circuits and particularly loss of synaptic plasticity, which have been implicated in depression. Regulation of glutamate homeostasis and promotion of healthy mitochondrial function are some of the physiological roles of LAC, and while altered LAC is a known biomarker of insulin resistance, LAC deficiency has also been recently reported in patients with MDD [20]. BDNF also play key roles in both metabolic homeostasis and brain areas involved in depression such as hippocampus [20]. PPAR-γ is a transcription factor abundantly expressed in adipose tissue where it serves as the chief regulator of adipogenesis and lipid metabolism, while also modulating whole-body insulin sensitivity [21]. Apart from its role in modulating peripheral insulin sensitivity, PPAR-γ pathway has also been linked with depression via its evidence-based involvement in neuroinflammation and neuroplasticity [20]. Moreover, insulin resistance and depressive disorders share similar lifestyle-related and behavioural risk factors such as physical inactivity, poor diet and sleep-wake cycle disturbances, among others. These factors contribute to allostatic load and overload, a vicious cycle of the body’s adaptive neural and systemic processes which eventually leads to increased systemic inflammatory responses, metabolic dysfunction and insulin resistance [20]. Collectively, these suggest that uncorrected insulin resistance may contribute to antidepressant treatment resistance in some depressed patients and that treatment response in this group may be enhanced by improving insulin sensitivity. Correction of insulin resistance may be achieved either pharmacologically or non-pharmacologically.

Non-pharmacological add-on therapy in depression may be beneficial in terms of treatment cost, side-effect profile and adherence. Some lifestyle and dietary-related interventions have been linked with improvement of insulin sensitivity in insulin-resistant and/or metabolic syndrome and/or type 2 diabetic patients. These include exercise [22, 23], healthy/Mediterranean diet (generally known to be rich in monounsaturated fatty acids) [24–27], hygienic/regulated sunlight exposure [28, 29], probiotics [30–32], magnesium supplementation [33, 34], vitamin D supplementation [35, 36] and zinc supplementation [37–41]. Although, at present, there is still controversy (due to conflicting results from relevant clinical studies) regarding the insulin sensitivity-enhancing effect of omega (n)-3 polyunsaturated acids (PUFA), the potential beneficial effect on insulin resistance has been linked with its well-known anti-inflammatory and triglycerides-lowering properties [42]. Indeed, higher omega-3 index has been found to be associated with increased insulin sensitivity and more healthy metabolic profile in some overweight men [43]. Apart from their effect on insulin sensitivity, some of the above-stated interventions have also shown beneficial effects in the treatment of depression. These include adjunctive exercise [44], vitamin D [45], magnesium [46] and zinc [47, 48] supplementation as well as use of n-3 PUFA [49] and probiotics [50–52]. Moreover, healthy diet, good sleep, physical activity and sunlight exposure, among others, have all been suggested as components of lifestyle medicine for depression [53]. The antidepressant effect of monounsaturated fatty acids (MUFA) has also been demonstrated in pre-clinical studies [54, 55]. Additionally, circadian rhythm disruption and sleep disturbances [56] involving too much [57], too little or poor quality [58, 59] sleep have been linked with impaired glucose metabolism. Worthy of note is the fact that sleep disorder (insomnia or hypersomnia) is one of the diagnostic symptoms of depression [60], with insomnia being more frequently reported [61]. Hence, evidence-based improvement in depressive symptoms with adjunctive sleep therapies such as behavioural and cognitive behavioural therapies as well as hygienic sleep habits (to treat insomnia and improve sleep quality, while also curtailing hypersomnia), may be linked with improvement in glucose homeostasis, especially in depressed patients with some level of insulin resistance.

A number of systematic reviews have shown that the above-stated lifestyle and dietary-related interventions improve depression outcomes when used as adjunctive or stand-alone treatments for depression [45, 47, 49, 50, 62]. A few trials have also reported improvement in metabolic and/or insulin sensitivity parameters alongside improvement in depression outcomes when these interventions are used as adjunctive therapy for depression [63–66]. However, no systematic review has been carried out to synthesise available evidence across all these studies with the aim of examining the potential role of the documented insulin sensitivity-enhancing effect of these adjuncts on antidepressant treatment improvement. Our hypothesis is that often undiagnosed and hence, uncorrected insulin resistance in patients with depression contributes to inadequate patient response to standard antidepressants and that adjunctive therapy with insulin sensitivity-enhancing lifestyle and dietary-related interventions may improve treatment response through correction of underlying insulin resistance. The main aim of this systematic review and meta-analysis is to evaluate the effectiveness of these non-pharmacological interventions in improving antidepressant treatment response, in comparison with adjunctive placebo or no adjuncts at all. This is with a view to testing the hypothesis stated above.

## Methods/design

### Type of studies

This systematic review will only include randomised controlled trials (RCTs) assessing the effect of insulin sensitivity-enhancing lifestyle and dietary-related adjuncts in clinically depressed patients.

### Inclusion criteria

#### Participants

Adults (aged ≥ 18 years) with a diagnosis of MDD, dysthymia or BD, based on International Classification of Diseases (ICD) [67], DSM-IV [68] or DSM-5 [60] criteria and experiencing depressive episode at baseline during the trials.

### Exclusion criteria

For the overall analysis of the effect of the interventions on the primary outcome, the following exclusion criteria apply:

- Trials in which participants had no standard diagnosis of clinical depression prior to enrolment or were not on standard antidepressant as TAU during the study protocol.
- Trials in which participants had other neurological or neuropsychiatric diagnosis, apart from depression in MDD, dysthymia or BD, or were taking other psychotropic medications apart from conventional antidepressants.
- Trials which included patients with other medical comorbidities apart from metabolic syndrome, obesity/insulin resistance or polycystic ovarian syndrome (PCOS).

### Interventions

We will consider specified types of lifestyle and dietary-related interventions having the potential to alter insulin sensitivity and used alongside conventional antidepressants (treatment as usual (TAU)), for subsequent comparison with the control groups described below. The duration of intervention had to have been a minimum of 4 weeks. This is because of the generally slow onset of action of conventional antidepressants and 4 weeks has been suggested to be the cut-off time point that predicts eventual response or remission after a longer duration of treatment [69]. The following types of adjunctive treatment will be included:

- Exercise: This involves engaging the participants in any form of structured and repetitive physical activity (as an add-on to TAU) over a defined period of time with the aim of improving general fitness, muscle and/or cardiovascular performance or health. There has to be a comparison group who were not offered this intervention and must be clearly stated by the authors as being the control for the exercise intervention group
- Vitamin D supplementation: Oral administration of vitamin D supplements at doses within the recommended dietary allowances (RDAs) and tolerable upper intake level for vitamin D in adults or other standard daily doses as might have been chosen and justified by the authors with the comparison group receiving placebo (with the TAU) instead of the dietary supplement and serving as the control.
- Zinc supplementation: Oral administration of zinc supplements at doses within the recommended dietary allowances (RDAs) and tolerable upper intake level for zinc in adults or other doses as chosen and justified by the authors with the comparison group similar to that described for vitamin D above.
- Magnesium supplementation: Oral administration of magnesium supplements with criteria similar to those described above for both zinc and vitamin D supplementation.
- Probiotics: Defined as live microorganisms which are similar to the gut flora and beneficial to health when taken in food or as dietary supplements. Trials in which probiotics were supplemented in any form (milk, yoghurt, capsule, powder) will be considered, with control requirements similar to that described above.
- Hygienic-dietary recommendations: A well-defined combination of sleep hygiene, healthy diet, moderate exercise/physical activity and sunlight exposure recommendations or any of these singly used (adjunctive to TAU) would be acceptable with a comparison group not being given any such strict recommendations/prescription and had to be stated by the authors as representing the control.
- Omega-3 polyunsaturated fatty acids: Oral administration of standard doses of fish oil or other omega-3 supplements, defined by the authors in comparison with placebo for the control group.
- Monounsaturated fatty acids: Oral administration of any type of clearly defined MUFA compound such as oleamide, in generally acceptable doses or MUFA-rich food (e.g. olive oil) in comparison with placebo (control group).

The antidepressants that will be considered as TAU should belong to any of the standard classes of conventional antidepressants including monoamine oxidase inhibitors (MAOIs), tricyclic antidepressants (TCAs), selective serotonin reuptake inhibitors (SSRIs), serotonin-noradrenaline reuptake inhibitors (SNRIs), noradrenaline reuptake inhibitors (NRIs) and miscellaneous standard antidepressants (e.g. Mirtazapine), all being monoamine-based antidepressants. Any of these antidepressants plus any mood stabiliser such as lithium (salts) will be considered as TAU in the treatment of depression pole in bipolar disorder.

### Comparison/control

Participants allocated to TAU alone or TAU + placebo to serve as control for subsequent comparison with the intervention groups.

### Outcomes

#### Primary outcomes

Remission and (significant) response, as pre-defined by the authors, in studies which reported binary outcome measures as well as improvement in depressive symptoms based on change in the standard depression rating scale scores (continuous variable) at the end of the treatment regimen. The following standard depression rating scales will be considered: Hamilton Rating Scale for Depression (HAM-D or HRSD), Beck Depression Inventory (BDI), Inventory of Depressive Symptomatology (IDS) or Montgomery-Asberg Depression Rating Scale (MADRS) [70].

#### Secondary outcome

Improvement in parameters of insulin resistance/sensitivity in studies that reported both baseline and follow-up (end of treatment regimen) assessment of one or more of the following:

○ Fasting plasma glucose (FPG)/fasting serum glucose (FSG)

○ Fasting plasma insulin/fasting serum insulin (FPI/FSI)

○ Oral glucose tolerance test (OGTT)

○ Homeostatic model assessment of insulin resistance and β-cell function (HOMA-IR, HOMA-B)

○ Quantitative insulin sensitivity check index (QUICKI) [71].

### Search strategy

#### Electronic searching

The search will be conducted on relevant bibliographic databases and trial registers. These include Cochrane Central Register of Controlled Trials (CENTRAL), MEDLINE, PubMed, Embase, PsychINFO as well as ClinicalTrials.gov and EU clinical trial register with publication dates restricted to 1 January 1990 to 30 November 2018. Also, only studies published in English language will be considered. In order to be able to capture as many studies as possible, the search will be based on two main concepts: the health condition in question (depression) and the different interventions of interest. On the databases, medical subject headings (MESH) or equivalent (e.g. Emtree, thesaurus) terms will be used when deemed best and free texts such as depress*[Title/Abstract], bipolar[Title/Abstract] and dysthmi* as well as different synonyms or variations of probiotics, exercise, vitamin D, magnesium, zinc supplementation, lifestyle, diet, unsaturated fatty acids, sunlight and sleep hygiene, will be used while also applying the ‘clinical trials’ and ‘publication date’ filters. These will then be logically set by using the Boolean operators ‘OR’ and ‘AND’ to combine the various descriptors within the same and different concepts respectively, in a systematic manner. An illustration of this is presented in Additional file 1.

#### Reference lists

The reference lists of included studies will be checked for identification of any other relevant articles, reviews and systematic reviews.

#### Grey literature

Authors of relevant studies might be contacted, if need be, to explore the possibility of being supplied with other data which are relevant but not readily accessible for the reason of being unpublished, informally published or components of ongoing studies. We will try to incorporate as much relevant data as possible in this systematic review.

#### Study identification and inclusion summary

The step-wise procedures involved in the identification, screening and inclusion of the relevant studies will be summarised using the preferred reporting items for systematic reviews and meta-analyses (PRISMA) flow chart/diagram [72]. This chart will also reflect the number of studies excluded and reasons for exclusion.

### Study selection

All citations identified by electronic searches will be downloaded to EndNote reference manager, with all duplicates removed. One reviewer (OJJ) will identify potentially relevant studies by reviewing titles and abstracts provided by the search. Full-text copies of all articles identified as potentially relevant will be obtained and independently assessed for inclusion by two reviewers (OJJ and BKR). Any disagreement regarding eligibility will be resolved by discussion and unanimous decision by all the review authors.

### Data extraction

Data will be independently extracted by two review authors (OJJ and BKR) using a pre-piloted data extraction form for collection of information on the following:

- Characteristics of participants: Mean age, sex, diagnosis and diagnostic system, number of participants, severity of depression at baseline, standard antidepressants in use (as TAU), study location and/or setting and metabolic comorbidities, if any.
- Details of intervention: Type, frequency and duration of intervention; randomisation, sample size in intervention and control arms as well as number analysed; withdrawals and/or drop-outs.
- Measures of outcome: % in remission and % that responded (if reported) as well as mean changes in depression scores (primary outcomes) for each group, as reported; mean changes in insulin sensitivity parameters (secondary outcome) for each group, as reported; reports of adverse effects of interventions, if any. For completeness, we will record the means and standard deviations in each treatment group at baseline and follow-up.

Any discrepancies in the data extraction information will be reconciled by involving all the review authors in discussion and a joint check through the original papers for adjudication by authors GC, FPL and BPK.

### Risk of bias assessment

A bias can be defined as a systematic error which often leads to a deflection from the truth (in either direction) in terms of results or inferences [73]. To assess the validity of our included studies, each trial will be assessed for risk of bias using the tools outlined in the Cochrane Handbook for Systematic Reviews of Interventions [73]. This involves assessment of bias risk across domains of sequence generation, allocation concealment, blinding (of participants, personnel and outcome assessors), incomplete outcome data (exclusion or attrition), selective outcome reporting and other sources of bias. Assessment across these six domains ensures that the risk of any of all the major types of bias in clinical trials (selection, performance, attrition, detection and reporting biases) are adequately addressed. The Cochrane ‘risk of bias’ table will be adapted which comprises of two main parts: (a) description of what was reported across each of the assessment domains, (b) answering pre-specified questions in relation to each of the specific entries, resulting in a judgement of ‘yes’ (adequate application and reporting of relevant criteria—low risk of bias), ‘no’ (relevant criteria was not/ineffectively applied—high risk of bias) and ‘unclear’ (insufficient detail to permit a definite judgement—unclear or uncertain risk of bias). An overall ‘risk of bias’ classification will then be assigned to each study, as follows:

- High risk of bias: One or more criteria not met
- Moderate risk of bias: Unclear judgement in one or more criteria; and
- Low-risk of bias: All criteria adequately met

All the review authors will be involved in the assessment and will not be blinded to the names of the authors, institutions, journals and results of the studies being assessed.

### Assessment of quality of evidence across studies

The quality of evidence is a reflection of the extent of confidence in the correctness of the estimate of the effect in question [74]. This will be assessed across the included studies using the Grading of Recommendations Assessment, Development and Evaluation (GRADE) system [75]. This system has four levels of classification, thus, high, moderate, low and very low to which each study can be assigned based on different factors starting from the study design. Evidence obtained from randomised controlled trials usually rates high at the start of the grading system but the confidence in such evidence may be decreased, due to factors such as study limitations (risk of bias), inconsistency of results, indirectness of evidence, imprecision and reporting/publication bias. These factors may eventually downgrade the overall quality of evidence from such studies which in turn happens to be a major factor for consideration in evaluating the strength of recommendations from the studies [75].

### Data synthesis and analysis

Synthesis of data for this review will be done by estimating the standardised mean difference (SMD) (for continuous variables) as a measure of the observed intervention effect for each included study. SMD is calculated by finding the mean difference in the values (change scores) of the continuous variables of interest (depression scores and insulin resistance parameters, in this meta-analysis) between the intervention and control groups at the end of the study and dividing it by the pooled standard deviation. SMD is a no-unit measure of effect size that allows for comparison of intervention effects across studies where the similar outcomes have been measured with different standard rating scales. Conventionally, SMD values of 0.2, 0.5 and 0.8 are taken as small, medium and large effect sizes, respectively [76]. Relative risks (RR) or odds ratios (OR) will also be calculated for dichotomous (binary) variables, if any. These will then be pooled together and analysed with random-effects (RE) meta-analysis model to obtain the summary effect estimate as well as 95% confidence interval and *p* value, using STATA or Review Manager (RevMan) software, version 5.3. Studies using binary and continuous outcomes will be combined separately. Outcomes will be presented on forest plots.

If feasible (depending on the data available), multivariate meta-analysis will be performed, incorporating both the primary and secondary outcomes in the analysis to evaluate the relationship between these outcomes [77]. This analysis will be performed using the mvmeta software in Stata [78].

Heterogeneity between studies will be explored through visual inspection of the forest plots and also using the *I*^2^ statistic which describes the percentage of total variation that is due to between-trials differences rather than chance (sampling error). A value of 0% is an indication of no observed inconsistency/heterogeneity while 0–25%, 25–50%, 50–75% and > 75% respectively indicate low, moderate, large and very large. Reasons behind any substantial (≥ 50%) heterogeneity will be explored by performing subgroup and sensitivity analyses, described below [79].

### Dealing with incomplete information

Where required study data is incomplete or clarifications are needed, authors of the studies will be contacted via e-mails (up to three times) during the study selection and data extraction process. Secondary publications of affected studies will also be sought for missing information. However, for studies where data are only available in graphic format, approximations of the mean will be imputed. In the instance of not being able to get the missing data with any of the above methods, estimation of standard deviations (SDs) will be done by borrowing SDs from other studies included in this meta-analysis [80].

### Subgroup analysis

- A major possible source of heterogeneity anticipated in this review is the presence of metabolic comorbidity (e.g. metabolic syndrome, insulin resistance, obesity) in some participants as opposed to others which will be evident from the assessment of baseline and follow-up parameters of glucose homeostasis. Hence, a subgroup analysis of studies which assessed these parameters will be performed for both the primary outcome and secondary outcome.
- If durations of included trials vary considerably, subgroup analyses will be carried out for comparison between short-duration and long-duration studies. Each included study will be regarded as having a short or long duration based on its duration relative to the median study duration of all the included studies, since a minimum (4 weeks) clinically meaningful duration for antidepressant treatment response is a criterion for inclusion and none of the included studies’ durations is expected to fall below this minimum.
- Depending on the number of included studies for each intervention type, subgroup analyses of the different intervention types will also be considered.
- Subgroup analyses based on the depression type (unipolar vs bipolar) will also be performed, subject to data availability.
- Additionally, subgroup analyses based on type of medication used (antidepressant vs antidepressant plus mood stabiliser) will be performed if the number of included studies suffice.

### Sensitivity analysis

To explore the effect of systematic errors (bias) across studies, sensitivity analysis will be carried out by excluding studies with high risk of bias, as classified under risk of bias assessment.

### Assessment of publication bias

If up to 10 trials are included in this meta-analysis, a funnel plot will be used to assess the risk of publication bias. The funnel plot will be assessed for asymmetry via visual inspection and Egger’s test [79] using the STATA statistical software.

### Presenting and reporting of results

The systematic review will be reported in accordance with the Preferred Reporting Items for Systematic Reviews and Meta-Analyses (PRISMA). The protocol will be registered with the International Prospective Registry of Systematic Reviews (PROSPERO) database (https://www.crd.york.ac.uk/prospero/).

## Discussion

The evidence-based association between depression and insulin resistance as well as the observed increased severity of depressive symptoms and the reported antidepressant ineffectiveness in patients with comorbid depression and insulin resistance warrant further investigation. This systematic review will qualitatively and quantitatively analyse trials assessing antidepressant treatment response following adjunctive insulin sensitising lifestyle and dietary-related interventions. These interventions include exercise, magnesium supplementation, vitamin D supplementation, zinc supplementation, probiotics, n-3 polyunsaturated fatty acids, monounsaturated fatty acids as well as hygienic-dietary recommendations which involves strict prescriptions of regulated daily exercise, healthy diet, hygienic sunlight exposure and hygienic sleep habits (combined in any form or any of these used as a stand-alone adjunct).

The antidepressant potential of these interventions has been separately linked with different mechanisms of action or neurobiological underpinning. For instance, exercise has been suggested to share a common mechanism of antidepressant action with fluoxetine as they were both found to increase neurogenesis and dendritic spine density in the hippocampi of mice [81]. This is further corroborated by studies which showed that exercise increases levels of brain-derived neurotrophic factor (BDNF) [82–84], a trophic factor known to promote functional and structural brain plasticity, hence associated with antidepressant effect. Similarly, the antidepressant effect of vitamin D has been associated with a number of mechanisms, including its role in promoting adult neurogenesis, catecholamine biosynthesis and amelioration of antioxidant injury to the brain, among others [85]. In addition to the involvement of zinc in modulating cytokine signalling and hippocampal neurogenesis, its antidepressant effect has also been attributed to its role in modulating the glutamatergic system [86] and monoaminergic system with a consequent impact on neural plasticity [87]. Magnesium, being a natural calcium antagonist, has been suggested to exert its antidepressant effect via its role in modulating glutamatergic neurotransmission. Studies have also demonstrated the involvement of serotonergic and noradrenergic systems in the antidepressant action mechanism of magnesium, as reviewed by Serefko et al. [88]. Magnesium has also been found to increase BDNF expression in the prefrontal cortex while also enhancing the activation of calcium/calmodulin-dependent protein kinase II (CaMKII) [88]. These together with its interaction with other factors that are key in the pathophysiology of depression such as suppression of hippocampal kindling have all been linked with the antidepressant action of magnesium [88]. In the same vein, the compelling evidence in support of the antidepressant potential of probiotics has been suggested to be through an increase in circulating serotonin induced by microbiome metabolites signalling and/or reduction of inflammatory responses across the gut-brain axis [52]. Furthermore, a number of inter-related mechanisms have been proposed as being associated with the potential antidepressant effect of the other lifestyle/behavioural interventions (healthy diet, sunlight exposure and sleep hygiene). Dietary habits modulate immune functions, oxidative processes, energy metabolism, neuroplasticity, among others, all of which are critical factors in the pathophysiological pathway of depression [89]. Epidemiological studies have shown an association between healthy dietary patterns (preferential consumption of more fruits, vegetables, whole grains, fish, nuts, etc.) and reduced incidence and prevalence of depression [90]. Fatty acids are known to play some roles in both depression [91] and insulin resistance [92]. Monounsaturated and n-3 polyunsaturated fatty acids, in particular, have both demonstrated beneficial (both preventive and therapeutic) effects in these two conditions. These effects have been consistently linked with their anti-inflammatory properties and other related mechanisms [92, 93]. Similar roles have also been associated with sleep; in fact, sleep, circadian rhythm and energy balance (hence, body weight) have been suggested to have parallel relationships via their intrinsic link with nutrient oxidation, ghrelin and leptin levels, appetite, food-reward homeostasis, hypothalamic-pituitary-adrenal (HPA) axis, with some of these also being critical factors in depression [94]. Although insomnia is more commonly reported in depression, hypersomnia/hypersomnolence is also often reported in some depressed patients, and the interaction between depression and hypersomnia is thought to be complex and often bidirectional [95]. Considering the pathophysiological pathways (such as those involving monoaminergic transmission) linking both insomnia and hypersomnia with depression, metabolic dysfunction and unhygienic lifestyle (e.g. poor diet and physical inactivity) alignment of sleep (and sleep architecture), feeding patterns (including food choices) and physical activity, as components of circadian alignment, is thought to be crucial for mood and metabolic functions. Apart from the established link between sunlight exposure and vitamin D synthesis/metabolism in the body, findings have suggested that light indirectly improves mood via the circadian timing system’s modulation of sleep homeostasis and also directly (independent of the circadian rhythm) via the intrinsically photosensitive retinal ganglion cells (ipRCGs)/melanopsin-expressing RCGs [96].

Despite the well-established insulin sensitivity-enhancing potential of these interventions, there has not been any previous comprehensive study or systematic review evaluating a possible link between their antidepressant effect and potential to correct insulin resistance in depressed patients. By analysing the antidepressant effect vis-à-vis the effect on glucose-handling parameters of lifestyle and dietary-related adjuncts collectively, this current review aims to evaluate the possibility of overcoming the problem of antidepressant ineffectiveness through correction of insulin resistance which when present is usually undiagnosed and hence uncorrected in some depressed patients.

This systematic review and analysis will be conducted in accordance with standard guidelines (as described above), minimising biases as much as possible while also discussing the strengths and limitations of the evidence that will be generated. Results that need to be interpreted with caution will be highlighted and used only for the purpose of hypothesis generation.

It is expected that this systematic review with meta-analysis will open a new ‘window’ regarding the potential basis for or mechanism involved in the antidepressant treatment response improvement observed with the use of the above-stated interventions as adjunctive therapy for depression. It will also inform future studies relating to potential new promises for interventions in the treatment of depression. Furthermore, this study will assist healthcare providers in making informed decisions relating to screening of depressed patients for insulin resistance and subsequent use of insulin sensitivity-enhancing interventions in patients with treatment-resistant depression and comorbid insulin resistance.

## Additional file

Additional file 1: Search strategy. (DOCX 12 kb)

## Abbreviations

BD: Bipolar Disorder
BDI: Beck Depression Inventory
DSM: Diagnostic and Statistical Manual of Mental Disorders
FPG: Fasting plasma glucose
FSG: Fasting serum glucose
HAM-D: Hamilton Depression Rating Scale
HOMA-B: Homeostatic model assessment of β-cell function
HOMA-IR: Homeostatic model assessment of insulin resistance
IDS: Inventory of Depressive Symptomatology
MADRS: Montgometry-Asberg Depression Rating Scale
MDD: Major depressive disorder
MDE: Major depressive episodes
MUFA: Monounsaturated fatty acids
OGTT: Oral glucose tolerance test
OR: Odds ratio
PDD: Persistent depressive disorder
PI: Plasma insulin
PUFA: Polyunsaturated fatty acids
QUICKI: Quantitative Insulin Sensitivity Check Index
RR: Relative risk
SD: Standard deviation
SI: Serum insulin
SMD: Standardised mean difference
TAU: Treatment as usual
