# Supplementary material for: Evaluation of the effect of insulin sensitivity-enhancing lifestyle- and dietary-related adjuncts on antidepressant treatment response: protocol for a systematic review and meta-analysis
Source: Syst Rev. 2019 Feb 25;8:62. doi: 10.1186/s13643-019-0978-8 (PMC6388470; doi:10.1186/s13643-019-0978-8)
Supplement: Supplementary file 1 — Search strategy. (DOCX 12 kb) [file 13643_2019_978_MOESM1_ESM.docx]

**Appendix:** **Search strategy**

In the preliminary search, the following search strategy was applied on PubMed. Same will be used for the review proper and adapted to other databases.

1. Depress*[Title/Abstract] Filters: Clinical Trial; Publication date from 1990/01/01 to 2018/11/30
2. Dysthymi*[Title/Abstract] Filters: Clinical Trial; Publication date from 1990/01/01 to 2018/11/30
3. Bipolar[Title/Abstract] Filters: Clinical Trial; Publication date from 1990/01/01 to 2018/11/30
4. Exercise[Title/Abstract] Filters: Clinical Trial; Publication date from 1990/01/01 to 2018/11/30
5. Physical activity[Title/Abstract] Filters: Clinical Trial; Publication date from 1990/01/01 to 2018/11/30
6. Lifestyle[Title/Abstract] Filters: Clinical Trial; Publication date from 1990/01/01 to 2018/11/30
7. Behaviour*[Title/Abstract] Filters: Clinical Trial; Publication date from 1990/01/01 to 2018/11/30
8. Behavior*[Title/Abstract] Filters: Clinical Trial; Publication date from 1990/01/01 to 2018/11/30
9. Vitamin D [MeSH Terms] Filters: Clinical Trial; Publication date from 1990/01/01 to 2018/11/30
10. Sunlight[Title/Abstract] Filters: Clinical Trial; Publication date from 1990/01/01 to 2018/11/30
11. Ultraviolet[Title/Abstract] Filters: Clinical Trial; Publication date from 1990/01/01 to 2018/11/30
12. Light[Title/Abstract] Filters: Clinical Trial; Publication date from 1990/01/01 to 2018/11/30
13. Diet*[Title/Abstract] Filters: Clinical Trial; Publication date from 1990/01/01 to 2018/11/30
14. Probiotics [MeSH Terms] Filters: Clinical Trial; Publication date from 1990/01/01 to 2018/11/30
15. Hygien*[Title/Abstract] Filters: Clinical Trial; Publication date from 1990/01/01 to 2018/11/30
16. Sleep [MeSH Terms] Filters: Clinical Trial; Publication date from 1990/01/01 to 2018/11/30
17. Zinc[Title/Abstract] Filters: Clinical Trial; Publication date from 1990/01/01 to 2018/11/30
18. Magnesium[Title/Abstract] Filters: Clinical Trial; Publication date from 1990/01/01 to 2018/11/30
19. ‘’Fatty acids, Unsaturated’’[Mesh] Filters: Clinical Trial; Publication date from 1990/01/01 to 2018/11/30
20. 1 OR 2 OR 3
21. 4 OR 5 OR 6 OR 7 OR 8 OR 9 OR 10 OR 11 OR 12 OR 13 OR 14 OR 15 OR 16 OR 17 OR 18 OR 19
22. 20 AND 21
